# Improving the state of health hardware in Australian Indigenous housing: building more houses is not the only answer

**DOI:** 10.3402/ijch.v72i0.21181

**Published:** 2013-08-05

**Authors:** Paul Pholeros, Tess Lea, Stephan Rainow, Tim Sowerbutts, Paul J. Torzillo

**Affiliations:** 1 Healthabitat, Newport Beach, Sydney, Australia; 2 Department of Gender and Cultural Studies, The University of Sydney, Sydney, Australia; 3 Q Social Research Consultants, Marrickville, Sydney, Australia; 4 Sydney Medical School, The University of Sydney, Sydney, Australia

**Keywords:** Indigenous housing, environmental health, Australia

## Abstract

**Background:**

This article outlines a program of applied research and development known as Housing for Health that, over the period 1999–2012, targeted health-related improvements in housing for Indigenous householders in communities across regional and remote Australia. In essence, the program focuses on measuring the functionality of key appliances and structures (we term this “health hardware”) against clear criteria and ensuring identified faults are fixed.

**Methods:**

Detailed survey and assessment of all aspects of housing was undertaken, particularly focusing on the function of health hardware. All results were entered into a database and analyzed.

**Results:**

The results demonstrate extremely poor initial performance of the health hardware. A key finding is that attention to maintenance of existing houses can be a cost-effective means of improving health outcomes and also suggests the need to superintend the health-conferring qualities of new infrastructure. We briefly outline the early foundations of the Housing for Health program, major findings from data gathered before and after improvements to household amenities, and our efforts to translate these findings into broader policy.

**Conclusions:**

These data demonstrate that simply injecting funds into housing construction is not sufficient for gaining maximum health benefit.

This article reports on the findings of an applied research and development program known as Housing for Health as it has been implemented across a range of Indigenous communities in Australia. As elaborated here, Housing for Health is a licensed survey-and-fix methodology that targets living conditions in community or public housing. We provide an update of early data (1) and share these findings with readers concerned with analogous environmental health issues in the circumpolar region.

Despite great differences in their cultural backgrounds and their internal heterogeneity, different Indigenous populations living in liberal settler countries tend to have worse health profiles than the non-Indigenous population across a range of measures (2–4). One explanation, based on the long-documented association between poor housing and poor health (5,6), is that poor living conditions are a key factor. Indigenous Australians not only share this health profile with other Indigenous peoples around the world, in some areas their morbidity and mortality status is worse. They have high rates of infectious diseases amongst children in addition to burgeoning chronic disease and early mortality amongst adults (4).

## Background

Although social and health commentary over the last three-quarters of a century have noted the association between the poor living environment and the poor health of Indigenous Australians, when the applied research and development program now known as Housing for Health began, there was little to no work that detailed the elements of the living environment that might contribute to poor health or that described the major health problems caused by such an environment. The work had to proceed from first principles.

The Housing for Health methodology evolved as an applied research and development project (1). The first pilot was commenced in Pipalyatjara, a small Aboriginal community in the Anangu Pitjantjatjara Yankunytjatjara Lands in an arid zone region of South Australia (7). The aim was to undertake a detailed study of the housing and living environment of Aboriginal people to isolate the environmental changes that would lead to maximum health gains, particularly for children aged 0–5 years. We developed a list of healthy living practices (HLPs) focussed around electrical safety, hygiene, and nutrition that would be necessary for anyone to keep themselves and their family well (1). We prioritized these according to their likely impact on health status, especially for young children. Electrical safety was the foundational priority, based on the risk of electrical fires that occur with sometimes fatal outcome for residents. Following electrical safety, the HLPs were:

1. Washing people
2. Washing clothes and bedding
3. Removing wastewater safely
4. Improving nutrition
5. Reducing overcrowding
6. Reducing the impact of animals, vermin, or insects
7. Reducing dust
8. Controlling temperature
9. Reducing trauma.

For each of these HLPs, we had basic objectives. For example, we considered that Aboriginal mothers should have access to the functioning health hardware (the equipment or “hardware” necessary to carry out these healthy living practices) to wash their children daily and to wash their hands and face frequently.

Having identified these practices, we then examined the kinds of health hardware—from toilets, lights, and drains to taps, showers, and kitchens.

In order to maintain good health among residents in relatively closed communities, the health hardware of most houses in those communities must function most of the time. Therefore, we also developed a means of consistently measuring whether this health hardware was in good working order. Because this could not be achieved through observations at a distance, and in order to enable measurement of longitudinal change, a range of standardized tests to assess the quality of housing was also piloted. These instruments were refined through processes of detailed and regular review as the program expanded to new sites across Australia (1). Throughout all projects, an emphasis on fixing faults that were identified through surveys—not just noting their prevalence—remained a core ethic. Survey teams were each issued a standardized toolbox so that any minor repairs not requiring a licensed tradesperson could be attended to immediately. This became known as the “survey–fix” process (1). Over time, as more Indigenous people and health professionals agitated for a Housing for Health project, a licensing system for operators was initiated, ensuring that the survey–fix methodology was implemented with consistency across different sites.

## Methods

The detailed methodology is described elsewhere (1) and summarized here. When communities agree to participate and funding for the work is secured, a Survey–Fix time is determined. During this period (often a week or more depending on community size), teams consisting of a trained and accredited team leader, community workers, and certified electricians and plumbers inspect, test, and record a total of up to 250 items in the houses and, where possible, undertake repair work at the time of assessment. Some items are assessed on a pass/fail basis against a cluster of criteria. For example, in assessing the ability of adults to wash, the shower facilities in houses are tested against seven essential features necessary for a shower to work in all seasons sustainably and on a reliable basis:

- Hot water delivered to shower
- Cold water delivered to shower
- Hot water temperature in acceptable range (>45°C, <62°C)
- Hot tap functioning
- Cold tap functioning
- Shower head functioning
- Shower drainage able to remove wastewater

All seven features need to be present and working for the house's shower facilities to be assessed as “passing.”

The information is recorded on survey sheets on the spot, with each house assessment taking approximately one hour to complete. During each day of Survey–Fix assessment, the collected data is entered on a database. In turn, the collated data generates a profile of tasks so that electricians and plumbers can immediately begin any corrective work requiring certified tradespersons. This stage of the project is known as Survey–Fix One (SF1). Once all houses in the community or survey area are assessed, a profile of major projects is developed. Within the limits of funding, these projects are subsequently attended to under supervision during the following six months. A second Survey–Fix program (SF2) is then undertaken to determine the degree of improvement in the health hardware performance of the assessed houses.

## Results

**Fig. 1:**
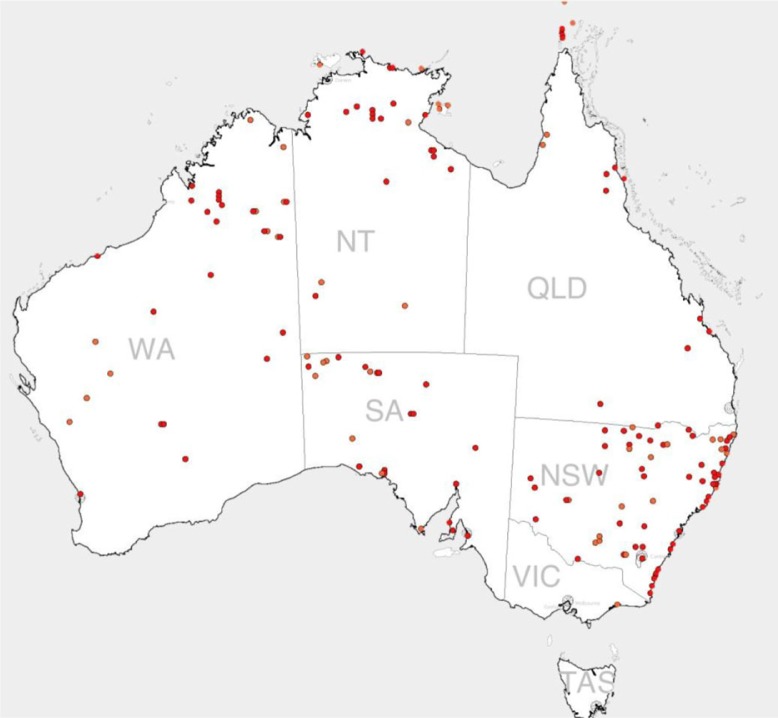
Map of community project sites, 1999–2012.

**Fig. 2:**
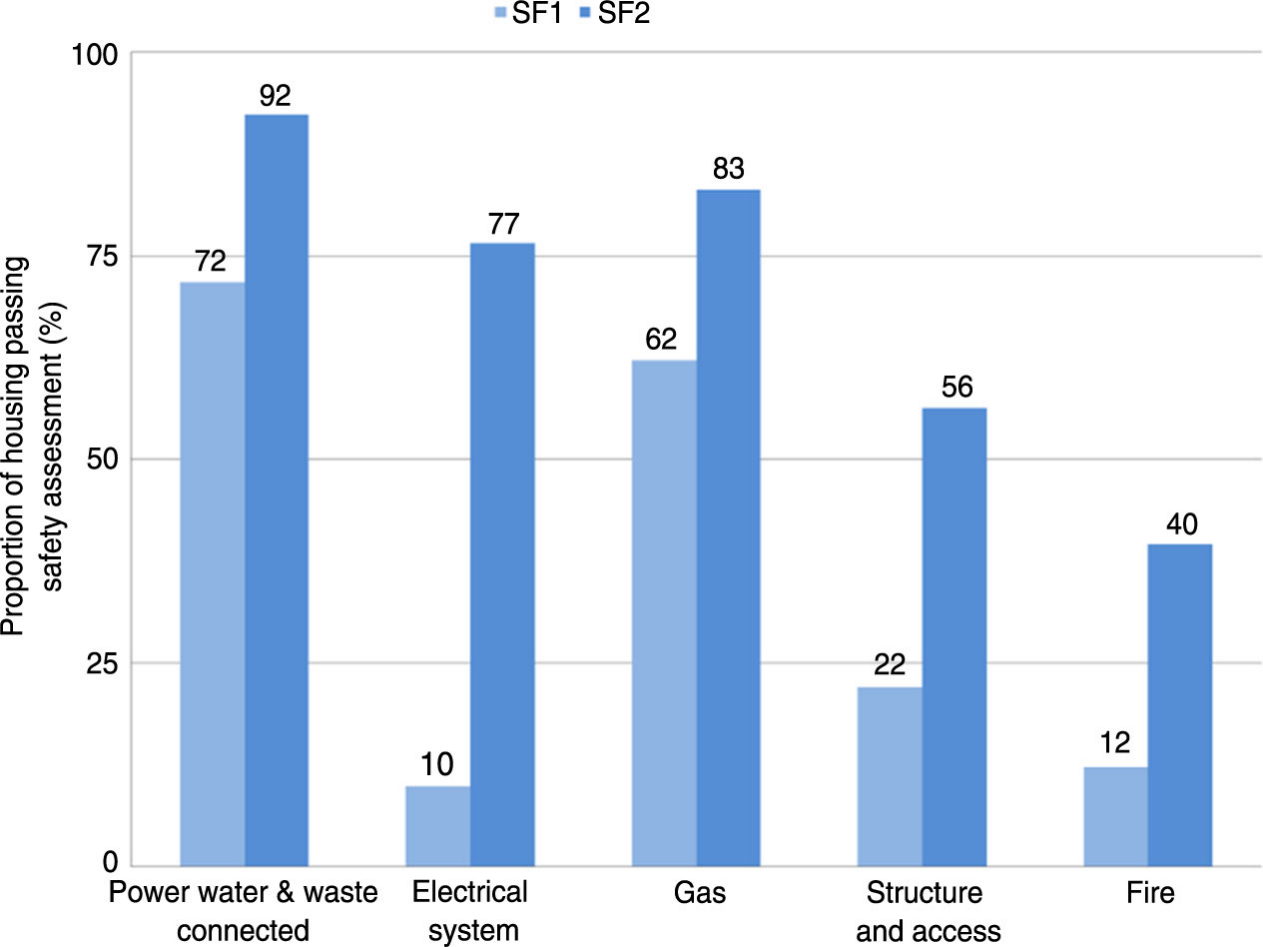
Housing for Health projects showing safety of 7,543 houses at Survey–Fix 1 before any fix work and 6,732 houses at Survey–Fix 2 after fix works; average cost per house $7,500.

**Fig. 3:**
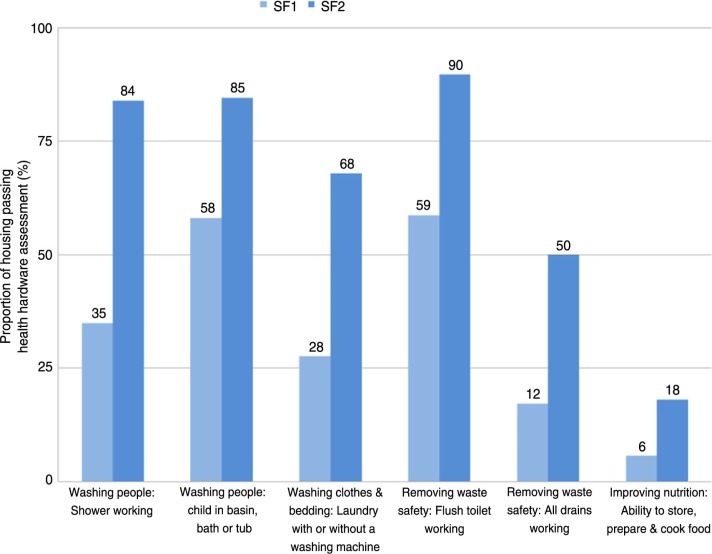
Housing for Health projects showing proportion of houses passing health hardware assessment in 7,543 houses at Survey–Fix 1 before any fix work and 6,732 houses at Survey–Fix 2 after fix works; average cost per house $7,500.

Between January 1999 and July 2012, 190 Aboriginal communities with 7,543 houses accommodating a population of approximately 45,000 people were assessed at SF1 and 6,732 houses at SF1 and SF2 using the Housing for Health licensed method. The distribution of communities is shown in Fig. 1. The data are presented in a manner that reflects the proportion of houses in which the initial Survey–Fix assessment (SF1) indicated safe functioning of particular aspects of the house (Fig. 2) or functioning health hardware in other hygiene or nutrition related domains (Fig. 3). Each of these tables also presents the degree of improvement at the SF2 following maintenance and repair work.

Seventy-eight percent of all people employed on the projects were local indigenous community members who were given training and basic equipment to undertake assessment of houses in their own communities.

Repairs by plumbers or electricians were required for 186,626 items, and these were categorized by these tradespersons as to the cause of failure. Nine percent of these items were the result of overuse/vandalism and the remainder were due to either a lack of routine maintenance (70%) or problems with initial construction and installation (21%).

## Discussion

These results demonstrate the extremely poor performance of Aboriginal housing in providing functioning health hardware. Only 10% of houses were electrically safe; 35% had a working shower; and 6% had a kitchen area in which food could be stored, prepared, and cooked. At SF2, there were substantial improvements in all areas of health hardware performance. The major limitation on improvement was funding. These projects had a per-house expenditure that depending on the organisational funding arrangements, ranged from $AUD 3,500 to $AUD 7,500. Since we prioritized electrical safety and hygiene, the greatest improvements were seen in these areas, and lesser degrees of improvement were seen in other domains. However, the results do demonstrate that substantial improvements in health hardware performance can be achieved with this model.

Supervision of initial construction and installation of equipment together with planned, funded, and delivered maintenance programs are essential to maintaining functioning health hardware. If these issues are not addressed in public housing programs, then simply building more houses is unlikely to improve the functioning health hardware necessary for people to sustain healthy living practices. Preliminary examination of data on health hardware performance scores of houses in different communities in one region (the Northern Territory) suggests that recently built or upgraded houses (within three years of assessment) do not perform better than older houses (data not shown). This again suggests that simply implementing new housing programs inadequately addresses the key issues of functioning health hardware and the need for well-supervised initial installation work and systematic maintenance programs.

Despite the clear indication that building new houses is no guarantee of improved environmental health, the Australian government continues to perceive this issue in terms of the huge investments required to build new houses. For example, the government committed $AUD 5.5 billion over a 10-year period commencing in 2008 to build 4,200 new houses at an average cost of $AUD 450,000 and to upgrade 4,800 houses at an average expenditure of $AUD 75,000. Although so far there has been no formal assessment of the health performance of the new houses built, our experience elsewhere with government-funded housing projects provides no reassurance that this massive expenditure in housing will actually improve health hardware function. Public housing reform in Indigenous Australia remains stymied by inconsistent maintenance and substandard materials used within key parts of the house and within its fabric.

Agitating for policy change by presenting data and logical case material in an attempt to persuade decision-makers and the “powers that be” to take a recommended course of action can only go so far (8). The relevant government minister is only partly responsible for what happens on the ground and cannot discuss the full range of interests that are simultaneously being balanced when housing and infrastructure projects are being plotted. In Australia, this fuller range of interests includes that of the construction industry and allied services, engineering companies, as well as the pastoral and mining industry sectors, and the costs of superintending works under contemporary government preferences for outsourcing regulation to private providers or permitting self-certification processes.

Our experience suggests that political commitment and financial expenditure on new capital works alone are not sufficient to address the detailed issues that link housing and health. This requires high-level technical advice; a strong commitment to design, supervision, and maintenance; and a mechanism to ensure a focus on functioning health hardware is sustained throughout all stages of such programs. It is in the bureaucratic oversight, management, and implementation of public housing programs where such technical advice is often disregarded. Simply put, policy formulators do not incorporate high quality supervision of works into their programming. It is wrongly assumed that contracting viable construction companies and self-certification processes will suffice to ensure that new and renovated housing meets national guidelines because the costs of independent and technically informed supervision for government contracted works are seen as too high. Longer-term costs are hidden within inevitable infrastructure decay (7,9). Some critical theorists have begun to refer to this as “infrastructural violence” and to draw attention to the difficulties of redressing systemic wrongs by trying to locate an individual culprit (a person or a policy) when a “faceless set of fleeting social connections” is a more accurate description of the planned and unplanned injustices that end up being inscribed into poorly functioning infrastructure (10).

The Housing for Health program has not been passive in terms of its policy advocacy. For instance, in order to help those responsible for implementing housing and infrastructure programs in Indigenous communities better understand what design and material considerations that need to be kept in mind, Housing for Health also initiated the *National Indigenous Housing Guide*, now in its third edition (11), and it successfully advocated for the HLPs to be integrated within government funded programs. However, all too often, this simply led to a cooption of our terminology and key components of the process were treated more cavalierly (8). In its most recent and most extensive public housing initiative, the government claimed its integration of the HLPs was so naturalized that they no longer needed to be specifically measured.

For these reasons, for those of us involved in directing Housing for Health into the future, appealing to the government by accumulating a huge amount of data on the legitimacy and worth of approaching maintenance systematically no longer holds appeal. Perhaps direct information sharing with the affected populations as well as those working in the field of social action via social media will be more effective.

## Conclusion

Our work has a number of implications for housing in Indigenous Australia and, we believe, for other groups living in public or community housing, including Indigenous nations such as those in the circumpolar region. Although climatic and geographic differences are large between Indigenous groups in different countries, the challenges share generic features. The details of design, construction, and maintenance will remain central to the health benefits of housing. The modern public health community tends to focus on research that measures health status in different housing environments. Although this approach is of interest, it may not lead to the health benefits desired unless there is a focus on the details of housing and health hardware and on how to prescribe their functionality. Housing and health literature has a long history (5,12) (13). It is replete with studies that have significant methodological problems with arguments usually centering on effect size; nevertheless, there is a strong signal that housing impacts both mental and physical health (14,15). It is important not to lose sight of the value of making change in the housing sector, an issue recognized more than a half century ago by Britten when he noted that “the inability to define the precise influence of the various elements of bad housing must not be an excuse for failing to make progress in improving housing conditions” (16) p. 193.
